# An Unexpected Case of Black Mamba (*Dendroaspis polylepis*) Bite in Switzerland

**DOI:** 10.1155/2017/5021924

**Published:** 2017-07-31

**Authors:** Verena Quarch, Lukas Brander, Luca Cioccari

**Affiliations:** Department of Intensive Care Medicine, Lucerne Cantonal Hospital, Lucerne, Switzerland

## Abstract

Mambas (genus* Dendroaspis*) are among the most feared venomous African snakes. Without medical treatment, mamba bites are frequently fatal. First-aid treatment includes lymphatic retardation with the pressure immobilization technique. Medical management comprises continuous monitoring, securing patency of the airway, ensuring adequate ventilation, symptomatic measures, and administration of specific antivenin. We report an unusual case of a snake breeder bitten by a black mamba in Switzerland, report the clinical course, and review the lifesaving emergency management of mamba bites. This case highlights the importance of early antivenin administration and suggests that emergency and critical care physicians as well as first responders all around the world should be familiar with clinical toxinology of exotic snake bites as well as with the logistics to most rapidly make the specific antivenin available.

## 1. Introduction

*Dendroaspis polylepis* (black mamba) is one of the most dangerous snakes worldwide. Without medical treatment, mamba bites are frequently fatal [1]. As mamba bites are rare in Europe [2–5] treatment can be challenging, particularly if rapid administration of antivenin fails [6]. We report the case of a Swiss snake breeder who was bitten by a black mamba, report the typical clinical course, and review the management of neurotoxic snake bites.

## 2. Case Presentation

Written informed consent for publication was obtained from the patient.

While feeding a 5-year-old male black mamba, a 34-year-old snake breeder suddenly noticed a tiny bloody mark on his forearm and, at the same time, a slight tingling of his lips. He immediately realized that he had been bitten and called a befriended snake expert to seek advice. The patient was thus able to provide the first responders with detailed information about the snake and on where to obtain the corresponding antivenin. He instructed his wife to apply a pressure bandage to the forearm. Within the next five minutes, chest tightness, generalized paresthesia, and fasciculation occurred. Upon arrival of the ambulance, the patient was unable to walk, was tachypneic, and had prominent dysarthria. Assuming a concomitant allergic reaction, the paramedics administered methylprednisolone, clemastine, and adrenaline before transferring the patient to the nearest hospital. In the meantime, the Swiss helicopter ambulance collected the antivenin from one of the 8 national antivenin depots.

Forty minutes after the bite, the patient arrived in the emergency department, complaining of worsening fasciculations and paresthesia affecting the extremities and the face. On physical examination, he was fully conscious with a heart rate of 105/min and a blood pressure of 165/80 mmHg. He was tachypneic at 30/min. Pulse oximetry revealed an oxygen saturation of 95% on room air. There were two tiny puncture wounds with local swelling and redness on the left forearm. Motor function was normal, except for mild ptosis. Seventy minutes after the bite, the patient was given 2 vials (20 ml) of “SAMIR Polyvalent Snake Antivenin” together with 2.5 mg of IV midazolam for ongoing hyperventilation. Thereafter, the patient was transferred to our tertiary intensive care unit for further treatment.

Upon arrival in our ICU, the patient was hemodynamically stable but still tachycardic and tachypneic. Fasciculations, dysarthria, and ptosis had slightly improved. ECG showed a grade 1 atrioventricular block without any other abnormalities. Initial laboratory tests were unremarkable, apart from moderate respiratory alkalosis. Over the next few hours, sweating, chills, and difficulty with swallowing as well as nausea occurred. However, the airway was never compromised, coughing reflex was intact, and respiratory failure did not occur. Therefore, and because of initial concerns about a possible allergic reaction, we decided against further antivenin administration. On the next day, symptoms of envenomation had improved, but the patient developed cellulitis of the bitten forearm and rhabdomyolysis, with a peak serum creatine kinase level of 16,049 U/L. Upon treatment with intravenous fluids and amoxicillin/sulbactam, his condition gradually improved. After four days in the hospital, he was discharged home with muscular pain as the only residual symptom. A few weeks later, the patient had fully recovered.

## 3. Discussion

Snake bites by* Dendroaspis* are rare in Europe. In 1987 Markwalder and Koller [2] described two cases of bites by* Dendroaspis viridis* (the green mamba). In France, one victim survived a green mamba bite although administration of antivenin failed [6]. Bites by black mambas have been reported in Germany [3] and in the Czech Republic [4]. To our knowledge, our case is the third registered black mamba bite in Switzerland and the first to be published.

The venom of black mambas is highly neurotoxic and contains a combination of *α*-neurotoxins, which induce postsynaptic blockade of the neuromuscular junctions, and dendrotoxins, which inhibit the voltage-dependent potassium channels, enhancing the release of acetylcholine at the neuromuscular junction, thus producing a neuromuscular block similar to a depolarizing block [7, 8]. In contrast, fasciculins act as acetylcholinesterase inhibitors, thus increasing the availability of acetylcholine at the neuromuscular junction and producing generalized, long-lasting fasciculations [9]. Calciseptine, another venom component, inhibits smooth muscle contraction and cardiac function by blocking L-type calcium channels [10]. The venom does not usually cause tissue destruction and necrosis [11, 12] as it lacks significant protease activity [13], although it does contain low percentages of other proteins, such as metalloproteinase, hyaluronidase, prokineticin, nerve growth factor, vascular endothelial growth factor, phospholipase A2, 5′-nucleotidase, and phosphodiesterase [7].

After a mamba bite, symptoms can occur as quickly as within 10 minutes [13]. A tingling sensation at the site of the bite may be the only initial sign of envenomation [14]. Other neurological symptoms include miosis, ptosis, blurred vision, bulbar symptoms, paresthesia, fasciculations, ataxia, and loss of consciousness. General signs of envenomation may include local pain, nausea, cough, and profuse sweating from sympathetic overstimulation. In severe cases, intubation, mechanical ventilation, and circulatory support may be necessary [4].

First-aid management includes reassuring the patient, removing constricting jewelry, and lymphatic retardation with pressure immobilization technique. Multiple bites are common, as mambas can strike repeatedly. A bandage is wrapped starting proximal to the bite site, just above the fingers or toes, and should cover the entire limb. Subsequent immobilization by a splint is recommended [15]. The bandage is not removed until administration of antivenin [16]. A tourniquet is not recommended.

The cornerstone of medical management is ensuring patent airway and adequate ventilation, providing circulatory support when necessary, and intravenous administration of specific antivenin [17]. Antivenin treatment should be considered whenever mamba envenomation is diagnosed by the presence of the systemic or neurological signs described above. Absence of fang marks does not preclude envenomation. On the other hand, presence of fang marks does not confirm it, since dry bites may occur. The recommended initial dose of SAIMR polyvalent antiserum is 20 ml (2 vials) [18]. Additional antivenin (up to five times the initial dose) should be titrated against the signs and symptoms of envenomation. Antivenin treatment is effective even when neurotoxic effects have become quite pronounced [19]. Therefore, there is no upper time limit for antivenin administration [20].

Antivenin therapy is not without risks. IgE-mediated allergic reactions including frank anaphylaxis can occur either to the antivenin or to the venom itself [21]. Delayed reactions (within 6–21 days after exposure) do occur, such as urticaria and serum sickness disease [22]. However, given the poor prognosis of an untreated mamba bite, even an anaphylactic response does not represent a contraindication to antivenin administration. In such cases, antivenin infusion should be temporarily discontinued and the patient should be stabilized before the antivenin infusion is resumed at a slower rate. An intravenous test dose of 1 ml diluted in 9 ml normal saline may be used in patients at high risk of allergic reactions. Limited evidence supports the use of prophylactic epinephrine prior to the administration of antivenins [23, 24]. There is no evidence for pretreatment with either antihistamines or corticosteroids [25, 26].

In our patient, fasciculation, muscle contractions, bulbar paralysis, and rhabdomyolysis were the main clinical symptoms. Respiratory failure did not occur for three possible reasons. First reason is the patient's immediate recognition of the bite and exemplary first-aid response, including pressure bandage and physical rest. Second, although the venom metering hypothesis is controversial [27] the amount of venom injected was probably submaximal as the bite was most likely defensive. Third, the rapid availability of antivenin prevented further deterioration, especially respiratory failure. However, the clinical course probably would have been more severe, if the patient himself had not reacted so swiftly. Our report highlights the importance of early antivenin administration. Emergency and critical care physicians as well as first responders around the world should, therefore, be familiar with clinical toxinology of snake bites and with the logistics to most rapidly make the specific antivenin available.
